# Comparative Study on Antistaphylococcal Activity of Lipopeptides in Various Culture Media

**DOI:** 10.3390/antibiotics6030015

**Published:** 2017-08-02

**Authors:** Maciej Jaśkiewicz, Damian Neubauer, Wojciech Kamysz

**Affiliations:** Department of Inorganic Chemistry, Faculty of Pharmacy, Medical University of Gdańsk, 80-416 Gdańsk, Poland; dneu@gumed.edu.pl (D.N.); kamysz@gumed.edu.pl (W.K.)

**Keywords:** antimicrobial peptides, lipopeptides, cyclic lipopeptides, *Staphylococcus aureus*, biofilm, culture media

## Abstract

*Staphylococcus aureus* bacteria are one of the leading microorganisms responsible for nosocomial infections as well as being the primary causative pathogen of skin and wound infections. Currently, the therapy of staphylococcal diseases faces many difficulties, due to a variety of mechanisms of resistance and virulence factors. Moreover, a number of infections caused by *S. aureus* are connected with biofilm formation that impairs effectiveness of the therapy. Short cationic lipopeptides that are designed on the basis of the structure of antimicrobial peptides are likely to provide a promising alternative to conventional antibiotics. Many research groups have proved a high antistaphylococcal potential of lipopeptides, however, the use of different protocols for determination of antimicrobial activity may be the reason for inconsistency of the results. The aim of this study was to learn how the use of various bacteriological media as well as solvents may affect activity of lipopeptides and their cyclic analogs. Obtained results showed a great impact of these variables. For example, cyclic analogs were more effective when dissolved in an aqueous solution of acetic acid and bovine serum albumin (BSA). The greater activity against planktonic cultures was found in brain-heart infusion broth (BHI) and tryptic-soy broth (TSB), while the antibiofilm activity was higher in the Mueller-Hinton medium.

## 1. Introduction

*Staphylococcus aureus* is one of the major pathogens responsible for variety of community- and hospital-acquired infections ranging from topical and harmless skin infections to severe systemic disorders such as endocarditis, necrotizing pneumonia, osteomyelitis or even sepsis [1,2]. Prevalence of staphylococcal diseases is related to the fact that some strains of the genus *Staphylococcus* such as *S. aureus* and *S. epidermidis* are typical skin colonizers. It has been determined that between 10% and 35% of healthy individuals are persistent carriers of *S. aureus*, and anterior nares are indicated as the most consistent sites from which these organisms can be cultured [3]. Moreover, there is a strong link between nasal carriage and increased risk of nosocomial infections [4]. Despite the fact that *S. aureus* and *S. epidermidis* are likely to belong to skin physiological flora, in some cases they can lead to opportunistic diseases. Moreover, those two strains have also been shown to be the most frequent causative factors of nosocomial infections on indwelling devices [5]. Treatment of staphylococcal diseases faces many difficulties, mainly because of a facile transmission (e.g., through exposure of the hands of healthcare workers), a variety of virulence factors, development of resistance to antibiotics, and the ability to adhere and form biofilm [4,6]. Biofilm can be defined as a microbial sessile community embedded within a self-produced matrix of extracellular polymeric substances that can be attached to biotic and abiotic surfaces. It seems likely that bacteria in some conditions prefer to form biofilm, firstly because extracellular matrix is capable of sequestrating and concentrating environmental nutrients, and secondly because it provides protection from exogenous stress factors such as antimicrobial substances or activity of immune system of invaded the organism. Another characteristic feature of the biofilm is a quorum-sensing. This term is referred to as the ability of bacteria to secrete low-molecular-weight signaling molecules that affect transcription patterns throughout local biofilm population. As a result of that activity, a better adaptation to rapid environmental changes is provided [7]. Since approximately 80% of all human bacterial infections are associated with biofilm, it is reasonable to consider antibiofilm activity of new antimicrobials [8]. Recently, various approaches to treat biofilm-related infections have been developed including antibodies, anti-adhesion strategies, the use of bacteriophages or quorum-sensing inhibitors [9]. Antimicrobial peptides (AMPs) are compounds with proven in vitro and in vivo antibiofilm activities [10]. These compounds are evolutionarily conserved molecules of the innate immune system of almost all organisms, playing an important role in warding off invading microbial pathogens [11]. To date, AMPs have been studied for their antimicrobial properties as they are targeting a broad spectrum of pathogens including bacteria, fungi, viruses, and protozoa [12,13]. Naturally occurring AMPs can vary in size and most of them do not have more than 50 amino acids in sequence. The majority are cationic molecules containing about 50% of hydrophobic amino acids [14]. Such properties allow them to electrostatically interact with a negatively charged bacterial surface, which leads to disruption of a cell membrane. Many attempts have been made to improve the activity and bioavailability of AMPs such as synthesis of short synthetic analogs, the use of D- or unnatural amino acids, fatty acid acylation or cyclization. Short cationic lipopeptides are compounds designed on the basis of AMPs amphiphilic character to imitate their antimicrobial properties. Net positive charge of these compounds as well as amphipathicity was obtained by combining the hydrophobic fragment of a fatty acid with a cationic peptide chain. The antimicrobial properties of short cationic lipopeptides have also been proven in several studies as a promising alternative for currently used antimicrobials [15,16]. Broth microdilution method is one of the leading procedures used in the in vitro susceptibility testing for bacteria, whereas the protocol issued by Clinical and Laboratory Standards Institute (CLSI) is being commonly used in scientific studies [17]. CLSI protocol is standardized and is internationally accepted by i.e., the European Committee on Antimicrobial Susceptibility Testing, the British Society for Antimicrobial Chemotherapy, the Deutsches Institut für Normung, and the Comité de l’Antibiogramme de la Société Française de Microbiologie. Owing to this assay the minimum inhibitory concentration (MIC) can be determined. However, since some peptides have a tendency to precipitate or bind to polystyrene, methods for evaluating their antimicrobial activity are being discussed. For instance, the most popular one was described by Hancock (University of British Columbia, Vancouver, BC, Canada) which involves different preparation of the peptide stock solution [18]. Despite the fact that there are reference protocols for MIC determination (which is actually conducted for planktonic forms of bacteria), there are no unified procedures used to determine antibiofilm activity. The aim of this study was to evaluate and to compare the results of antistaphylococcal activity (against both planktonic and biofilm forms) of short cationic lipopeptides and their cyclic analogs in three different microbiological media: Mueller Hinton Broth (which is used in CLSI and Hancock protocol), Brain-Heart Infusion Broth, and Tryptic Soy-Broth. Stock solutions of lipopeptides were prepared in both PBS and aqueous solution of BSA and AcOH.

## 2. Results

Lipopeptides used in this study are based on previously described compounds with high antistaphylococcal activity (i.e., Pal-KKKK-NH_2_, Pal-RRR-NH_2_) [16,19,20]. All of the lipopeptides are palmitoylated at N-terminus and contain three or four basic amino acid residues. Moreover, compounds are amidated at C-termini to maximize positive charge. Arginine residue was introduced at different positions to learn if this simple procedure may provide more active compounds. Arginine, as a basic amino acid, introduce positive charge; however, it has more hydrogen donor atoms and is more basic (pKa~12.5) than lysine (pKa~10.5). Interestingly, plenty of naturally occurring lipopeptides are cyclic. This fact inspired us to design cyclic analogs and evaluate the relevance of disulfide cyclization in case of antimicrobial activity of simple short cationic lipopeptides. In this study, each linear lipopeptide has its cyclic counterpart with a intramolecular disulfide bridge where cationic amino acid residues are placed between two cysteine residues. As a result, all compounds have well defined hydrophobic and hydrophilic regions. The identity of all compounds was confirmed by LC-MS analysis (ESI-MS) and all mass spectra are included in the supplementary materials.

**Figure 1:**
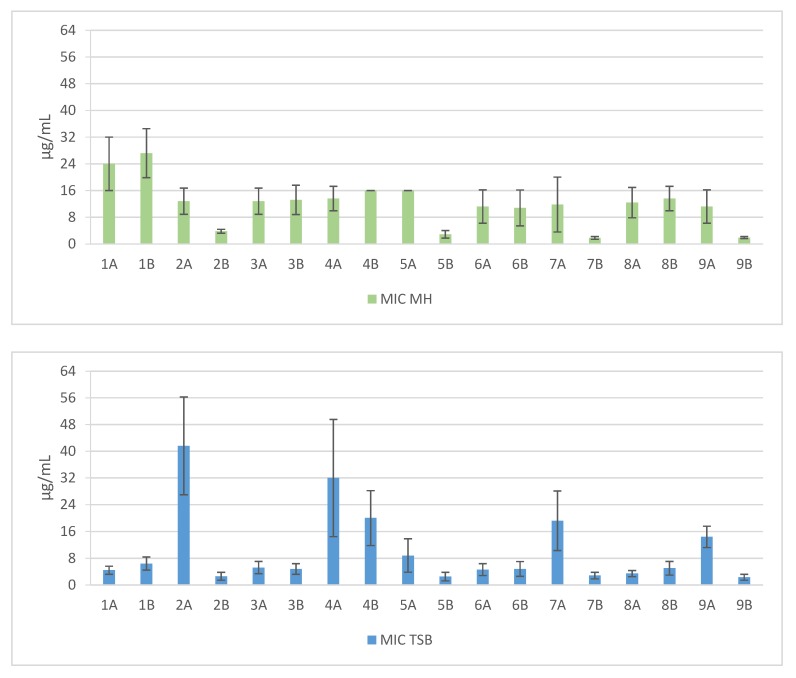

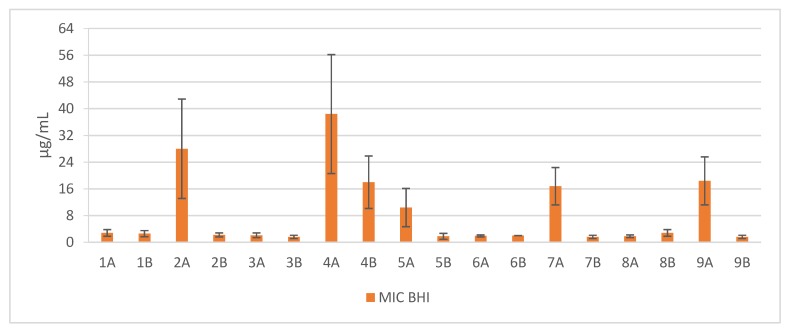
Mean MIC values against clinical and reference strains of *Staphylococcus aureus*. 1—Pal-KKK-NH_2_; 2—Pal-CKKKC-NH_2_; 3—Pal-KKKR-NH_2_; 4—Pal-CKKKRC-NH_2_ (linear); 5—Pal-CKKKRC-NH_2_; 6—Pal-KKRK-NH_2_; 7—Pal-CKKRKC-NH_2_; 8—Pal-RKKK-NH_2_; 9—Pal-CRKKKC-NH_2_ (cyclic); Solvents: A—PBS; B—AcOH/BSA.

**Figure 2:**
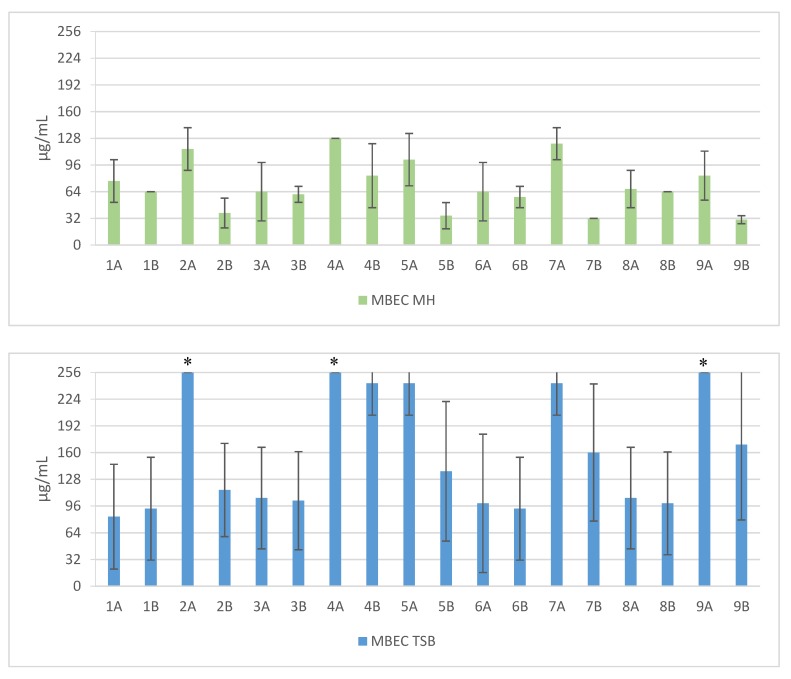

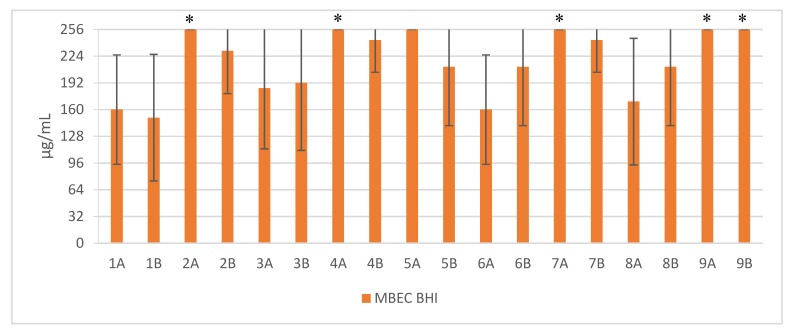
Mean MBEC values against clinical and reference strains of *Staphylococcus aureus*. 1—Pal-KKK-NH_2_; 2—Pal-CKKKC-NH_2_; 3—Pal-KKKR-NH_2_; 4—Pal-CKKKRC-NH_2_ (linear); 5—Pal-CKKKRC-NH_2_; 6—Pal-KKRK-NH_2_; 7—Pal-CKKRKC-NH_2_ (cyclic); 8—Pal-RKKK-NH_2_; 9—Pal-CRKKKC-NH_2_; Solvents: A—PBS; B—AcOH/BSA; *—concentration > 256 µg/mL.

Short cationic lipopeptides and their cyclic analogs were active against all tested strains of *S. aureus.* However, the antistaphylococcal activity depended on the type of medium as well as the solvent used for compound solubilization. MIC values for linear lipopeptides were lower in the BHI and TSB media than in MH (Figure 1). It was only in the case of non-cyclized Pal-CKKKRC-NH_2_ that this dependence was not observed. In the case of cyclic lipopeptides, the influence of medium type was irrelevant (Pal-CKKRKC-NH_2_ and Pal-CRKKKC-NH_2_ were slightly more active in MH). Despite this fact, all cyclic compounds dissolved in acetic acid/BSA were as a rule more active in comparison to those dissolved in PBS. The antibiofilm activity was highest in the MH medium (Figure 2) and cyclic analogs were as active as the linear forms or twofold more active (but only with compounds dissolved in BSA/acetic acid solution). Interestingly, the activity of cyclic lipopeptides was related to the medium and stock solution type. The antibiofilm activity improved in the order MH > TSB > BHI. Moreover, cyclic lipopeptides were even fourfold more active when dissolved in the AcOH/BSA solution. With linear lipopeptides, the type of stock solution seems to be less relevant. Biofilms formed in BHI exhibited resistance to almost all tested compounds, while in the case of TSB medium, cyclic analogs dissolved in PBS did not eradicate the staphylococcal biofilm.

## 3. Discussion

Such comprehensive research on antimicrobial activity of lipopeptides has not yet been conducted. In this study, many factors were taken into account, including the type of microbiological medium, the use of various peptide stock solution solvents, and the impact of lipopeptide cyclization as well. Moreover, the verification of the activity conducted on 10 strains of *S. aureus* (5 reference and 5 clinical ones) revealed a significant difference in antimicrobial activity of the lipopeptides in miscellaneous microbiological media. The reason of this difference may be associated with environment-dependent plasticity of lipid composition of *S. aureus* membranes. Sen et al. (2016) proved that the membranes of staphylococci grown in MH were less fluid than cells grown in BHI or TSB, which was related to a higher content of branched-chain fatty acids (BCFAs) and carotenoids [21]. These findings may be much more interesting as the staphyloxanthin, a carotenoid pigment, is one of the most potent virulence factors of *S. aureus* [22]. Staphyloxanthin provides integrity of the cell membrane and improves the resistance to reactive oxygen species this enhancing bacterial survival [23,24,25]. Furthermore, the influence of carotenoid content on susceptibility to host defense peptides was also investigated. In an in vitro study conducted by Mishra et al. (2011), the carotenoid-overproducing strains were less susceptible to daptomycin and cationic antimicrobial peptides such as human neutrophil defensin-1 (hNP-1), platelet microbicidal proteins (PMPs), and polymyxin B [26]. These results are consistent with the findings that higher carotenoid concentrations in *S. aureus* cell membrane cultivated in MH medium might suppress the activity of the tested compounds. In our study, the susceptibility of *S. aureus* biofilms in various growth media was examined. Biofilms formed in the BHI and TSB mediums were insensitive toward lipopeptides. The reason for this situation might be related to the impact of the glucose content on biofilm formation. In contrast to the MH medium (starch 1.50 g/L), BHI and TSB are supplemented with d-glucose (2.00 g/L and 2.50 g/L, respectively). Some studies proved the role of glucose in adhesion induction and modulation of biofilm formation [27,28]. Waldrop et al. (2014) have found that the mass of *S. aureus* biofilms rises with an increasing concentration of glucose with a threshold at 2.00–2.40 g/L [29]. Moreover, the catabolite control protein A (CcpA), which is responsible for regulation of various staphylococcal virulence determinants and resistance to antibiotics, is also regulated by the presence of the glucose [30]. Another factor that may have an impact on the activity of antimicrobials is the salt content of the media. BHI and TSB, in contrast to MH, are supplemented with NaCl (5.0 g/L) and this difference should be taken into account. In fact, the antistaphylococcal activity of some antibiotics such as aminoglycosides or oxacillin decreases in a concentration-dependent manner [31,32]. Nevertheless, our previous studies (2016) on the influence of several salts on antimicrobial activity of lipopeptides showed no difference in the MIC values against *S. aureus* strains for the wide range of NaCl concentration. Moreover, among all tested salts, only sodium acetate and sodium trifluoroacetate were found to increase the activity of tested compounds [33]. Different pH values of the media may also be crucial for the antimicrobial properties. However, the impact of pH differences may not be relevant in the case of BHI, MH and TSB since these culture media have almost the same pH (7.4, 7.4 and 7.3, respectively).

It should be noted that conclusions drawn from the in vitro studies are not always consistent with those determined in vivo. Nevertheless, these “not-ideal” conditions may reveal an unexpected activity of new compounds. For example, the study conducted by Citterio et al. (2016) showed the synergy between human plasma and antimicrobial peptidomimetics, AMPs, and antibiotics against several bacterial pathogens [34]. In this case, the MIC values decreased down to sixteenfold in the presence of plasma. The synergistic effect may be connected with complement proteins or clotting factors, as the activity of all compounds in heat-inactivated plasma were significantly decreased. Another factor included in our study was the use of various solvents for peptide stock solution. Interestingly, lipopeptides dissolved in an aqueous solution of acetic acid and bovine serum albumin (compounds marked with the letter B) were generally more effective than those dissolved in PBS. This approach was supported by a hypothesis that the type of solvent used in stock solution might have an impact on peptide solubility even after serial dilution in culture medium. For example, Steinberg et al. (1997) conducted a study on antimicrobial properties of protegrin-1 using a reference protocol issued by CLSI (formerly NCCLS) and modified a method wherein the peptide was prepared in an aqueous solution of acetic acid and BSA. Interestingly, the MIC values obtained using this modification were significantly lower; 5-times against *S. aureus* and *Enterococcus faecalis* and up to 30-times against *Escherichia coli* and *Pseudomonas aeruginosa* [35]. Similar observations were reported by Giacometti et al. (2000), who compared several methods for the determination of the activity of AMPs such as buforin II, cecropin, indolicidin, magainin II, nisin, and ranalexin [18]. Generally, our observation is inconsistent with the previously determined impact of BSA which is known to reduce lipopeptide antibacterial activity due to strong binding of fatty acids [36,37]. However, in our study, the concentration of BSA was relatively low and thus lipopeptides remained active. BSA may have a negative influence on the antimicrobial activity of lipopeptides and in our study its application gives even better results than PBS stock solution; this shows that phosphate ion should be considered as an activity suppressor, especially in the case of cyclic lipopeptides. Hypothetically, rigid cyclic structure and amino acids side chain orientation promote electrostatic interactions between amine and guanidine moieties with phosphate ion. Cyclization of AMPs is a popular approach to improve the activity and/or selectivity of the parent molecule [38]. For example, in research conducted by Unger et al. (2001), the cyclization of melittin analogs resulted in an increased antibacterial activity. Interestingly, the cyclization also resulted in decreased hemolytic properties; this suggests a lower toxicity towards mammalian cell lines [39]. It has been shown that disulfide cyclization may result in enhanced peptide activity, selectivity, and stability [40]. In our study, cyclic lipopeptides were found to be more effective against all tested staphylococci, but only if they were dissolved in an aqueous solution of acetic acid and BSA. Interestingly, the cyclization did not improve the antibiofilm activity in BHI or TSB. Nevertheless, it remains difficult to indicate which method provides more reliable data. Moreover, different positioning of arginine residue along the peptide chain of both linear and cyclic lipopeptides seems to be irrelevant for antistaphylococcal activity. Despite the fact that protocols utilized for the determination of antimicrobial activity are standardized, the influence of different factors such as solvents for stock solutions or culture media used for in vitro studies may play a pivotal role in selecting promising antimicrobial compounds; this is especially true when taking into account the activity against biofilm. This study indicated the relevance of each variable, despite the overall chemical similarities between lipopeptides.

## 4. Materials and Methods 

### 4.1. Synthesis of Lipopeptides

**Figure 3:**
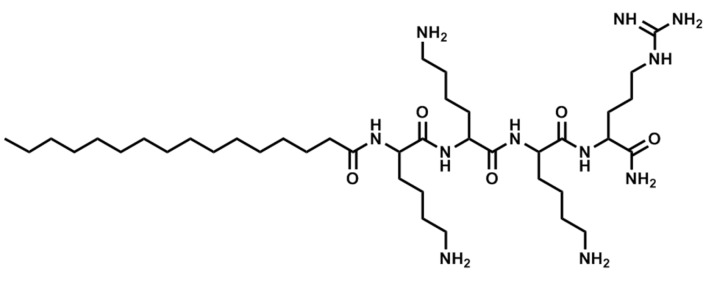
Structure of Pal-KKKR-NH_2_.

**Figure 4:**
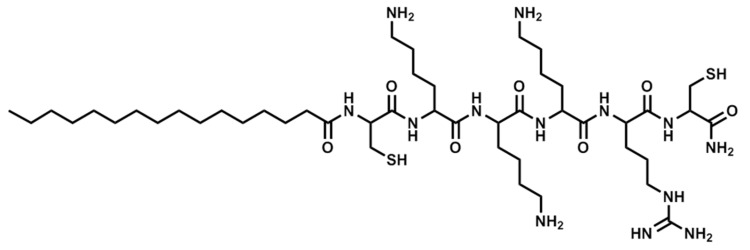
Structure of Pal-CKKKRC-NH_2_ (linear).

**Figure 5:**
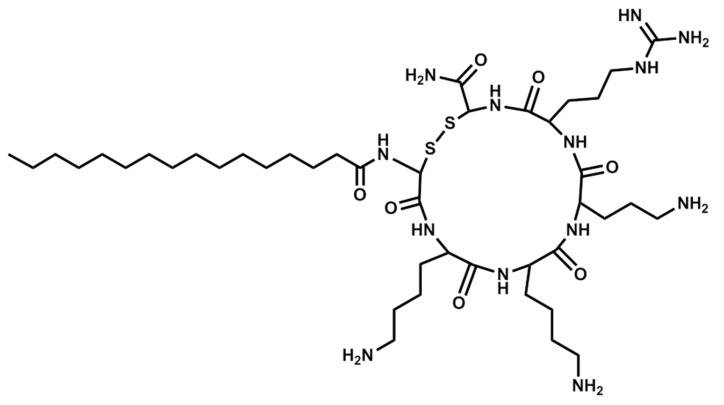
Structure of Pal-CKKKRC-NH_2_ (cyclic).

The compounds were obtained by manual synthesis on solid support (polystyrene AM-RAM resin) using Fmoc/t-Bu strategy. Coupling reactions were carried out in DCM and DMF mixture using DIC as a coupling reagent and OxymaPure as racemisation suppressant. All reagents were used in fourfold excess based on the resin. Deprotection was performed with 20% piperidine solution in DMF for 15 m. Cleavage from the resin was accomplished in TFA and scavengers mixture—TFA:TIS:water, 96:2:2 (*v*/*v*) for 90 m with agitation. Additionally, EDT was applied as a scavenger if the peptide contained cysteine residues. Cyclic analogs were obtained by intramolecular disulphide bridge formation through oxidation with iodine. The lipopeptides were purified using RP-HPLC. The purity and identity of the peptides were confirmed by LC-MS analysis. The structures of selected lipopeptides used in the study are presented in Figure 3, Figure 4 and Figure 5.

### 4.2. Antimicrobial Assays

#### 4.2.1. MIC Assay

Antimicrobial activity was determined against five reference strains: *S. aureus* ATCC 25923, *S. aureus* ATCC 6538, *S. aureus* ATCC 6538/P, *S. aureus* ATCC 9144, and *S. aureus* ATCC 12598, which were obtained from the Polish Collection of Microorganisms (Polish Academy of Sciences, Wroclaw), and against five clinical strains of *S. aureus*, which were provided by the Department of Oral Microbiology, Medical University of Gdańsk (Poland). These strains were also characterized in terms of methicillin resistance. Preliminary identification and detection was conducted on the ChromID MRSA/ChromID *S. aureus* biplate (bioMérieux) for simultaneous detection of *S. aureus* and methicillin-resistant *S. aureus* (MRSA). All tested strains (both reference and clinical *S. aureus*) were stored in cryo-vials (Roti^®^-Store, Carl Roth) and recultivated before all microbiological tests. All strains were cultured in three different media: Brain-Heart Infusion Broth (BHI, Biocorp, Warsaw, Poland), Mueller Hinton Broth (MH, Biocorp), and Tryptic-Soy Broth (TSB, Biocorp). Moreover, the stock solutions of lipopeptides were prepared in two different solvents: PBS and an aqueous solution of acetic acid/BSA (0.01% and 0.2% respectively). The complete list of compounds used in this study and their signatures are presented in Table 1. MIC was determined by broth microdilution method on polystyrene 96-well plates. Bacteria at initial inoculums of 5 × 10^5^ CFU/mL were exposed to lipopeptides at concentrations ranging from 0.5 to 256 µg/mL and incubated for 18 h at 37 °C. The MIC value was taken as the lowest concentration of the compound that inhibited the growth of the microorganisms. All assays were performed in triplicate and included the growth and sterility control.

#### 4.2.2. MBEC Assay

*S. aureus* biofilms were cultured on polystyrene 96-well flat bottom plates. Bacteria at initial inoculum of 5 × 10^8^ CFU/mL in BHI, MH or TSB were added to the plates and incubated at 37 °C. After 24 h of incubation, all *S. aureus* cultures were rinsed three times with PBS (pH 7.4) and a fresh medium was added. Subsequently, all biofilms were exposed to lipopeptides at concentrations ranging from 0.5 to 256 µg/mL for 24 h at 37 °C. After incubation, resazurin (7-hydroxy-3*H*-phenoxazin-3-one 10-oxide, Sigma Aldrich, Steinheim, Germany) was added as a cell viability reagent and the MBEC was read. All assay were performed in triplicate and included the growth and sterility control.

## Figures and Tables

**Table 1:** List of lipopeptides used in this study.

Sequence	Structure	Solvent	Signature
Pal-KKK-NH_2_	Linear	PBS	1A
Pal-KKK-NH_2_	Linear	AcOH/BSA	1B
Pal-CKKKC-NH_2_	Cyclic	PBS	2A
Pal-CKKKC-NH_2_	Cyclic	AcOH/BSA	2B
Pal-KKKR-NH_2_	Linear	PBS	3A
Pal-KKKR-NH_2_	Linear	AcOH/BSA	3B
Pal-CKKKRC-NH_2_	Linear	PBS	4A
Pal-CKKKRC-NH_2_	Linear	AcOH/BSA	4B
Pal-CKKKRC-NH_2_	Cyclic	PBS	5A
Pal-CKKKRC-NH_2_	Cyclic	AcOH/BSA	5B
Pal-KKRK-NH_2_	Linear	PBS	6A
Pal-KKRK-NH_2_	Linear	AcOH/BSA	6B
Pal-CKKRKC-NH_2_	Cyclic	PBS	7A
Pal-CKKRKC-NH_2_	Cyclic	AcOH/BSA	7B
Pal-RKKK-NH_2_	Linear	PBS	8A
Pal-RKKK-NH_2_	Linear	AcOH/BSA	8B
Pal-RKKK-NH_2_	Cyclic	PBS	9A

## Supplementary Materials

The following are available online at http://www.mdpi.com/2079-6382/6/3/15/s1, Table S1: MIC values [µg/mL] of Staphylococcus aureus strains (SA) in Mueller-Hinton Broth (MH), Table S2: MIC values [µg/mL] of Staphylococcus aureus strains (SA) in Brain-Heart Infusion Broth (BHI), Table S3: MIC values [µg/mL] of Staphylococcus aureus strains (SA) in Tryptic Soy Broth (TSB), Table S4: MBEC values [µg/mL] of Staphylococcus aureus strains (SA) in Mueller-Hinton Broth (MH), Table S5: MBEC values [µg/mL] of Staphylococcus aureus strains (SA) in Brain-Heart Infusion Broth (BHI), Table S6: MBEC values [µg/mL] of Staphylococcus aureus strains (SA) in Tryptic Soy Broth (TSB), Table S7: Measured and calculated m/z of lipopeptides, Figure S1: Mass spectrum of Pal-KKK-NH_2_, Figure S2: Mass spectrum of Pal-CKKKC-NH_2_, Figure S3: Mass spectrum of Pal-RKKK-NH_2_, Figure S4: Mass spectrum of Pal-CRKKKC-NH_2_, Figure S5: Mass spectrum of Pal-KKRK-NH_2_, Figure S6: Mass spectrum of Pal-CKKRKC-NH_2_, Figure S7: Mass spectrum of Pal-KKKR-NH_2_, Figure S8: Mass spectrum of Pal-CKKKRC-NH_2_ (cyclic), Figure S9: Mass spectrum of Pal-CKKKRCNH_2_ (linear).

## Abbreviations

The following abbreviations are used in this manuscript:

AcOH	Acetic acid
AMPs	antimicrobial peptides
BSA	Bovine serum albumin
CFU	Colony forming unit
CLSI	Clinical and Laboratory Standards Institute
DCM	Dichloromethane
DIC	*N*,*N*’-diisopropylcarbodiimide
DMF	*N*,*N*-dimethylformamide
EDT	1,2-ethanedithiol
Fmoc	9-fluorenylmethoxycarbonyl
MBEC	Minimum biofilm eradication concentration
MIC	Minimum inhibitory concentration
Pal	Palmitic acid
PBS	Phosphate buffer saline
TIS	Triisopropylsilane
TFA	Trifluoroacetic acid
