# Supplementary material for: Comparative Study on Antistaphylococcal Activity of Lipopeptides in Various Culture Media
Source: Antibiotics (Basel). 2017 Aug 2;6(3):15. doi: 10.3390/antibiotics6030015 (PMC5617979; doi:10.3390/antibiotics6030015)
Supplement: Supplementary file 1 [file antibiotics-06-00015-s001.pdf]

Supplementary Materials: Comparative Study on Antistaphylococcal Activity of Lipopeptides in Various Culture Media

Maciej Jaśkiewicz, Damian Neubauer and Wojciech Kamysz

Table S1. MIC values [μg/mL] of *Staphylococcus aureus* strains (SA) in Mueller-Hinton Broth (MH).

Compound	SA 25923	SA 6538	SA 6538/P	SA 9144	SA 12598	SA 1N*	SA 1S*	SA 2N	SA 2S	SA 3N
PAL-KKK (PBS)	32	16	32	16	16	32	32	16	16	32
PAL-KKK (ACOH/BSA)	32	16	32	16	16	32	32	32	32	32
PAL-CKKKC (PBS)	8	8	16	16	8	8	16	16	16	16
PAL-CKKKC (ACOH/BSA)	4	2	4	4	4	4	4	4	4	4
PAL-KKKR (PBS)	16	8	16	16	8	16	8	8	16	16
PAL-KKKR (ACOH/BSA)	16	8	8	16	4	16	16	16	16	16
PAL-CKKKRC lin. (PBS)	16	8	16	8	8	16	16	16	16	16
PAL-CKKKRC lin. (ACOH/BSA)	16	16	16	16	16	16	16	16	16	16
PAL-CKKKRC (PBS)	16	16	16	16	16	16	16	16	16	16
PAL-CKKKRC (ACOH/BSA)	4	1	4	2	2	4	4	2	2	4
PAL-KKRK (PBS)	16	4	8	16	4	8	16	8	16	16
PAL-KKRK (ACOH/BSA)	16	4	4	16	4	8	8	16	16	16
PAL-CKKRKC (PBS)	8	2	8	8	4	8	32	16	16	16
PAL-CKKRKC (ACOH/BSA)	2	1	2	2	1	2	2	2	2	2
PAL-RKKK (PBS)	16	8	4	16	8	8	16	16	16	16
PAL-RKKK (ACOH/BSA)	16	8	16	16	8	8	16	16	16	16
PAL-CRKKKC (PBS)	8	8	16	4	4	8	16	16	16	16
PAL-CRKKKC (ACOH/BSA)	2	2	2	2	2	1	2	2	2	2

N—strain isolated from anterior nares; S—strain isolated from skin; *—MRSA strain.

Table S2. MIC values [µg/mL] of *Staphylococcus aureus* strains (SA) in Brain-Heart Infusion Broth (BHI).

Compound	SA 25923	SA 6538	SA 6538/P	SA 9144	SA 12598	SA 1N*	SA 1S*	SA 2N	SA 2S	SA 3N
PAL-KKK (PBS)	4	2	4	4	2	2	2	2	2	4
PAL-KKK (ACOH/BSA)	2	2	2	4	4	2	4	2	2	2
PAL-CKKKC (PBS)	8	32	16	16	16	32	32	32	32	64
PAL-CKKKC (ACOH/BSA)	2	2	2	2	2	4	2	2	2	2
PAL-KKKR (PBS)	2	2	4	2	2	1	2	2	2	2
PAL-KKKR (ACOH/BSA)	1	2	1	2	2	2	2	1	1	2
PAL-CKKKRC lin. (PBS)	32	64	64	64	32	32	32	16	16	32
PAL-CKKKRC lin. (ACOH/BSA)	4	16	16	16	16	16	32	16	16	32
PAL-CKKKRC (PBS)	4	8	4	4	4	16	16	16	16	16
PAL-CKKKRC (ACOH/BSA)	2	2	2	2	4	1	2	1	1	1
PAL-KKRK (PBS)	2	2	2	2	2	1	2	2	2	2
PAL-KKRK (ACOH/BSA)	2	2	2	2	2	2	2	2	2	2
PAL-CKKRKC (PBS)	16	16	16	16	8	16	32	16	16	16
PAL-CKKRKC (ACOH/BSA)	2	1	2	2	2	2	2	1	1	1
PAL-RKKK (PBS)	2	2	2	2	2	2	2	1	2	1
PAL-RKKK (ACOH/BSA)	2	2	2	4	4	2	4	2	4	2
PAL-CRKKKC (PBS)	16	16	16	16	8	16	32	16	16	32
PAL-CRKKKC (ACOH/BSA)	2	1	1	2	1	2	2	1	2	2

N—strain isolated from anterior nares; S—strain isolated from skin; *—MRSA strain.

Table S3. MIC values [µg/mL] of *Staphylococcus aureus* strains (SA) in Tryptic Soy Broth (TSB).

Compound	SA 25923	SA 6538	SA 6538/P	SA 9144	SA 12598	SA 1N*	SA 1S*	SA 2N	SA 2S	SA 3N
PAL-KKK (PBS)	4	4	4	8	4	4	4	4	4	4
PAL-KKK (ACOH/BSA)	8	4	8	8	8	4	8	4	4	8
PAL-CKKKC (PBS)	32	64	64	32	64	32	32	32	32	32
PAL-CKKKC (ACOH/BSA)	4	4	4	2	4	2	2	1	1	2
PAL-KKKR (PBS)	4	8	4	4	8	4	8	4	4	4
PAL-KKKR (ACOH/BSA)	8	4	4	4	4	4	8	4	4	4
PAL-CKKKRC lin. (PBS)	32	64	64	32	32	16	32	16	16	16
PAL-CKKKRC lin. (ACOH/BSA)	16	32	16	16	16	32	32	16	8	16
PAL-CKKKRC (PBS)	4	8	4	4	4	8	16	8	16	16
PAL-CKKKRC (ACOH/BSA)	4	2	4	4	4	1	2	1	1	2
PAL-KKRK (PBS)	4	4	4	4	4	4	8	2	8	4
PAL-KKRK (ACOH/BSA)	8	4	4	8	4	8	4	2	4	2
PAL-CKKRKC (PBS)	32	16	8	16	8	16	32	16	16	32
PAL-CKKRKC (ACOH/BSA)	4	2	2	4	2	4	2	2	4	2
PAL-RKKK (PBS)	2	4	4	4	2	4	4	4	2	4
PAL-RKKK (ACOH/BSA)	2	4	4	4	4	4	8	8	4	8
PAL-CRKKKC (PBS)	16	16	8	16	16	16	16	8	16	16
PAL-CRKKKC (ACOH/BSA)	4	2	2	2	2	2	2	1	4	2

N—strain isolated from anterior nares; S—strain isolated from skin; *—MRSA strain.

Table S4. MBEC values [µg/mL] of *Staphylococcus aureus* strains (SA) in Mueller-Hinton Broth (MH).

**Compound**	**SA 25923**	**SA 6538**	**SA 6538/P**	**SA 9144**	**SA 12598**	**SA 1N***	**SA 1S***	**SA 2N**	**SA 2S**	**SA 3N**
PAL-KKK (PBS)	64	64	128	64	128	64	64	64	64	64
PAL-KKK (ACOH/BSA)	64	64	64	64	64	64	64	64	64	64
PAL-CKKKC (PBS)	64	128	128	64	128	128	128	128	128	128
PAL-CKKKC (ACOH/BSA)	16	16	32	32	32	32	32	64	64	64
PAL-KKKR (PBS)	32	64	128	32	128	32	64	64	64	32
PAL-KKKR (ACOH/BSA)	64	64	64	64	64	32	64	64	64	64
PAL-CKKKRC lin. (PBS)	128	128	128	128	128	128	128	128	128	128
PAL-CKKKRC lin. (ACOH/BSA)	32	64	64	128	128	32	64	64	128	128
PAL-CKKKRC (PBS)	64	128	128	64	128	64	64	128	128	128
PAL-CKKKRC (ACOH/BSA)	16	32	32	16	32	32	32	32	64	64
PAL-KKRK (PBS)	32	64	128	32	128	64	32	32	64	64
PAL-KKRK (ACOH/BSA)	64	64	64	64	64	64	32	32	64	64
PAL-CKKRKC (PBS)	128	128	128	64	128	128	128	128	128	128
PAL-CKKRKC (ACOH/BSA)	32	32	32	32	32	32	32	32	32	32
PAL-RKKK (PBS)	64	64	128	32	64	64	64	64	64	64
PAL-RKKK (ACOH/BSA)	64	64	64	64	64	64	64	64	64	64
PAL-CRKKKC (PBS)	128	128	128	64	64	64	64	64	64	64
PAL-CRKKKC (ACOH/BSA)	32	32	32	32	32	32	16	32	32	32

N—strain isolated from anterior nares; S—strain isolated from skin; *—MRSA strain.

Table S5. MBEC values [μg/mL] of *Staphylococcus aureus* strains (SA) in Brain-Heart Infusion Broth (BHI).

**Compound**	**SA 25923**	**SA 6538**	**SA 6538/P**	**SA 9144**	**SA 12598**	**SA 1N***	**SA 1S***	**SA 2N**	**SA 2S**	**SA 3N**
PAL-KKK (PBS)	128	128	128	128	128	128	>256	>256	64	>256
PAL-KKK (ACOH/BSA)	32	128	>256	128	64	128	>256	128	128	>256
PAL-CKKKC (PBS)	>256	>256	>256	>256	>256	>256	>256	>256	>256	>256
PAL-CKKKC (ACOH/BSA)	128	>256	>256	>256	128	>256	>256	>256	>256	>256
PAL-KKKR (PBS)	128	128	>256	128	64	128	>256	>256	>256	>256
PAL-KKKR (ACOH/BSA)	64	>256	>256	>256	64	128	>256	>256	128	>256
PAL-CKKKRC lin. (PBS)	>256	>256	>256	>256	>256	>256	>256	>256	>256	>256
PAL-CKKKRC lin. (ACOH/BSA)	128	>256	>256	>256	>256	>256	>256	>256	>256	>256
PAL-CKKKRC (PBS)	>256	>256	>256	>256	>256	>256	>256	>256	>256	>256
PAL-CKKKRC (ACOH/BSA)	64	>256	>256	>256	128	128	>256	>256	>256	>256
PAL-KKRK (PBS)	128	128	128	128	64	128	>256	>256	128	>256
PAL-KKRK (ACOH/BSA)	>256	128	>256	>256	64	>256	>256	>256	128	>256
PAL-CKKRKC (PBS)	>256	>256	>256	>256	>256	>256	>256	>256	>256	>256
PAL-CKKRKC (ACOH/BSA)	>256	>256	>256	>256	128	>256	>256	>256	>256	>256
PAL-RKKK (PBS)	32	128	128	128	128	128	>256	>256	>256	>256
PAL-RKKK (ACOH/BSA)	64	>256	>256	>256	128	128	>256	>256	>256	>256
PAL-CRKKKC (PBS)	>256	>256	>256	>256	>256	>256	>256	>256	>256	>256
PAL-CRKKKC (ACOH/BSA)	>256	>256	>256	>256	>256	>256	>256	>256	>256	>256

N—strain isolated from anterior nares; S—strain isolated from skin; *—MRSA strain.

Table S6. MBEC values [µg/mL] of *Staphylococcus aureus* strains (SA) in Tryptic Soy Broth (TSB).

**Compound**	**SA 25923**	**SA 6538**	**SA 6538/P**	**SA 9144**	**SA 12598**	**SA 1N***	**SA 1S***	**SA 2N**	**SA 2S**	**SA 3N**
PAL-KKK (PBS)	32	128	>256	64	64	64	64	64	32	64
PAL-KKK (ACOH/BSA)	64	64	>256	32	64	64	64	128	128	64
PAL-CKKKC (PBS)	>256	>256	>256	>256	>256	>256	>256	>256	>256	>256
PAL-CKKKC (ACOH/BSA)	128	64	>256	64	128	128	64	128	64	128
PAL-KKKR (PBS)	32	128	>256	128	64	64	64	128	64	128
PAL-KKKR (ACOH/BSA)	64	64	>256	64	128	64	64	128	64	128
PAL-CKKKRC lin. (PBS)	>256	>256	>256	>256	>256	>256	>256	>256	>256	>256
PAL-CKKKRC lin. (ACOH/BSA)	128	>256	>256	>256	>256	>256	>256	>256	>256	>256
PAL-CKKKRC (PBS)	>256	>256	>256	>256	>256	>256	>256	128	>256	>256
PAL-CKKKRC (ACOH/BSA)	128	64	>256	32	>256	128	64	>256	64	128
PAL-KKRK (PBS)	64	32	>256	128	64	64	32	>256	32	64
PAL-KKRK (ACOH/BSA)	64	64	>256	32	64	64	64	128	64	128
PAL-CKKRKC (PBS)	>256	>256	>256	128	>256	>256	>256	>256	>256	>256
PAL-CKKRKC (ACOH/BSA)	>256	64	>256	64	128	>256	64	>256	128	128
PAL-RKKK (PBS)	64	32	>256	128	128	64	128	128	64	64
PAL-RKKK (ACOH/BSA)	32	64	>256	64	64	64	64	128	128	128
PAL-CRKKKC (PBS)	>256	>256	>256	>256	>256	>256	>256	>256	>256	>256
PAL-CRKKKC (ACOH/BSA)	>256	64	>256	32	128	128	64	>256	>256	>256

N—strain isolated from anterior nares; S—strain isolated from skin; *—MRSA strain.

MS analysis

ESI-MS, Positive scan, Range: 50.00-1250.00 Da; Cone voltage 10V

Table S7. Measured and calculated m/z of lipopeptides.

Compound	Average mass	z	Calculated m/z	Measured m/z
Pal-KKK-NH_2	639.97	1	640.97	640.69
		2	320.99	321.01
		3	214.33	214.39
Pal-CKKKC-NH_2	844.24	1	845.25	844.70
		2	423.13	423.04
		3	282.42	282.43
Pal-RKKK-NH_2	796.15	1	797.16	796.84
		2	399.08	399.14
		3	266.39	266.59
Pal-CRKKKC-NH_2	1000.43	1	1001.44	1000.83
		2	501.22	501.19
		3	334.45	334.62
Pal-KKRK-NH_2	796.15	1	797.16	796.80
		2	399.08	399.24
		3	266.39	266.53
Pal-CKKRKC-NH_2	1000.43	1	1001.44	1000.77
		2	501.22	501.11
		3	334.45	334.45
Pal-KKKR-NH_2	796.15	1	797.16	796.81
		2	399.08	399.11
		3	266.39	266.42
Pal-CKKKRC-NH_2 (cyclic)	1000.43	1	1001.44	1000.80
		2	501.22	501.11
		3	334.45	334.51
		4	251.12	251.11
Pal-CKKKRC-NH_2 (linear)	1002.44	1	1003.45	1002.86
		2	502.23	501.86

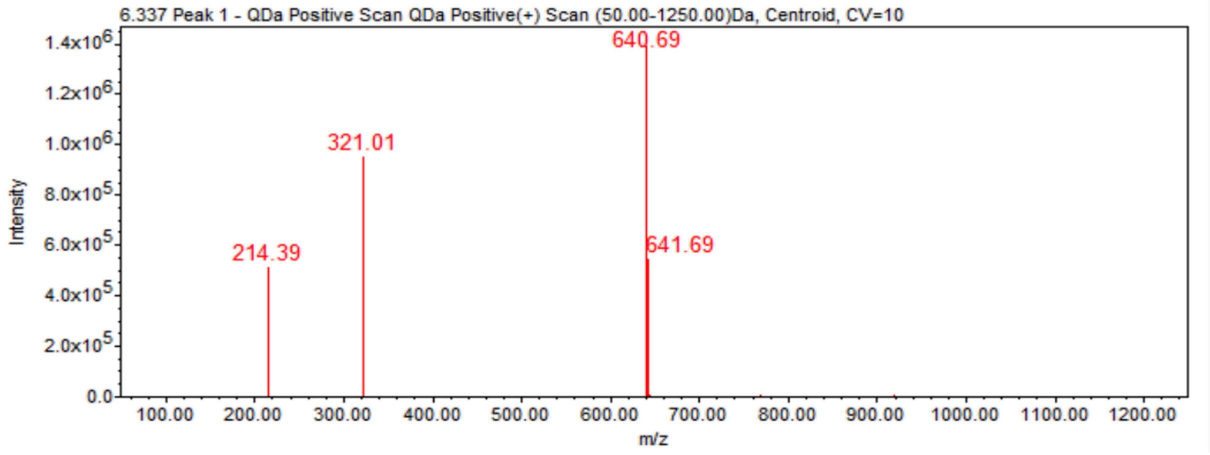

Figure S1. Mass spectrum of Pal-KKK-NH2.

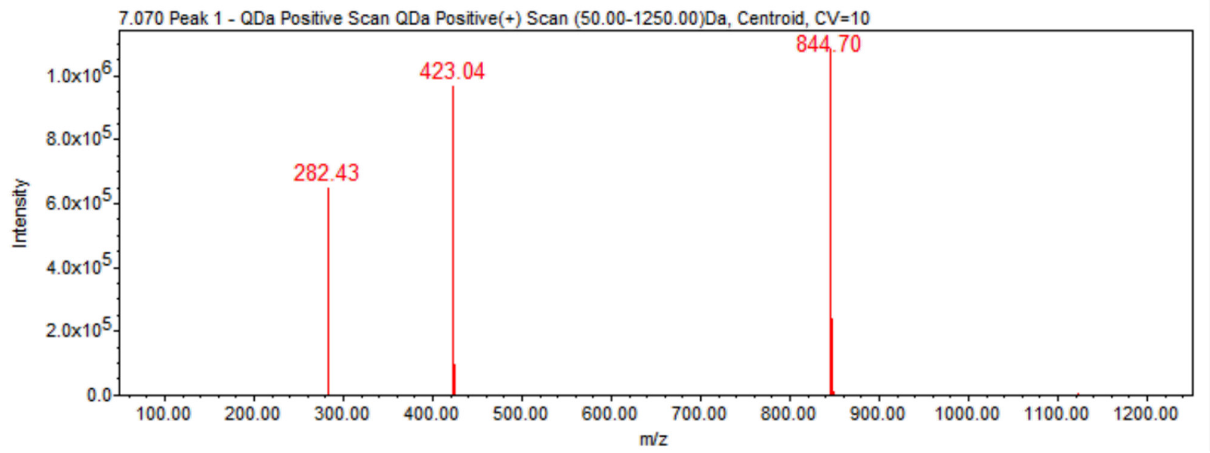

Figure S2. Mass spectrum of Pal-CKKKC-NH2.

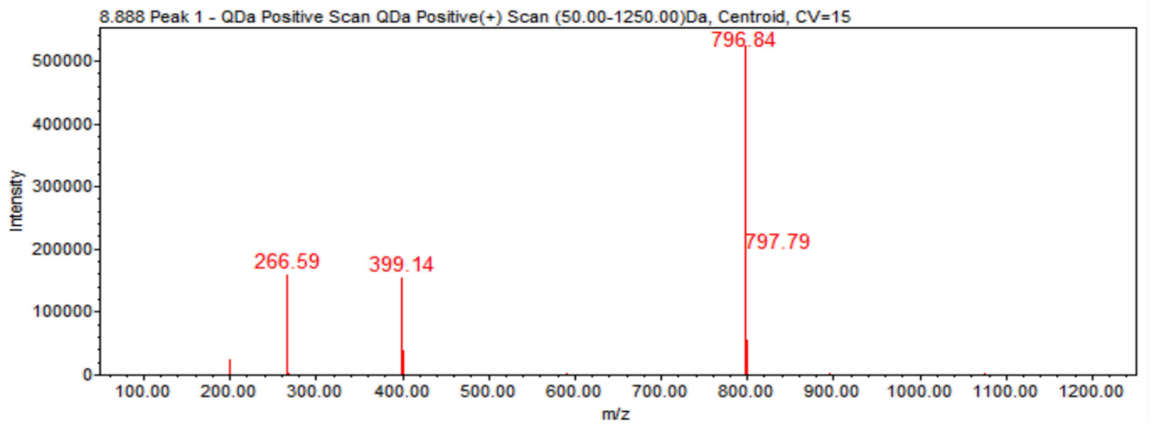

Figure S3. Mass spectrum of Pal-RKKK-NH_2.

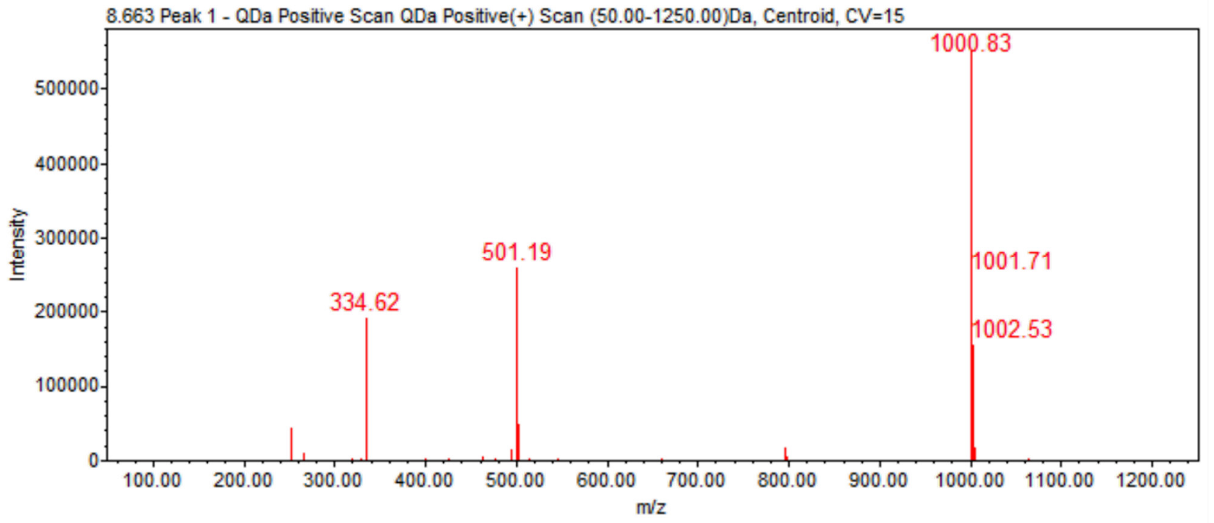

Figure S4. Mass spectrum of Pal-CRKKKC-NH_2.

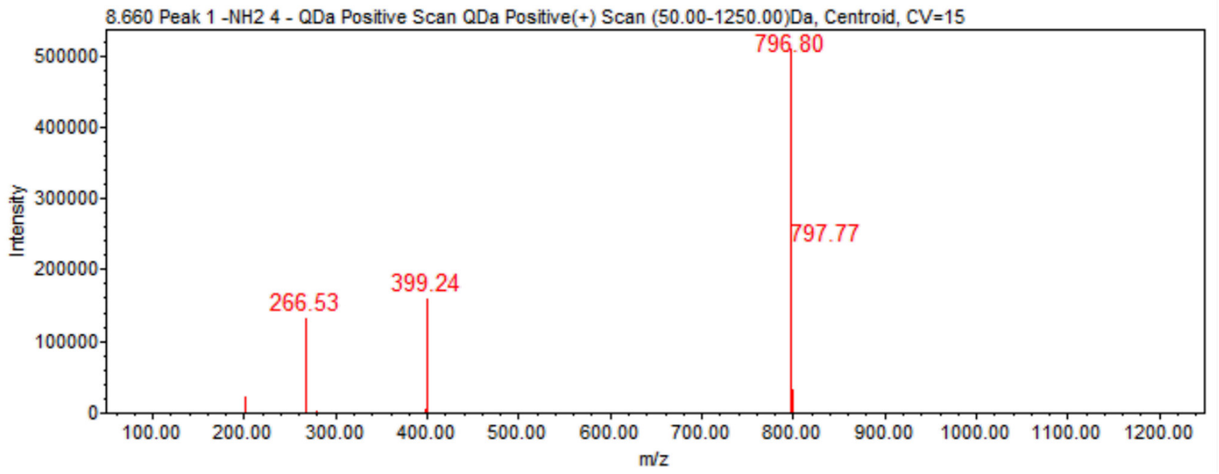

Figure S5. Mass spectrum of Pal-KKRK-NH_2.

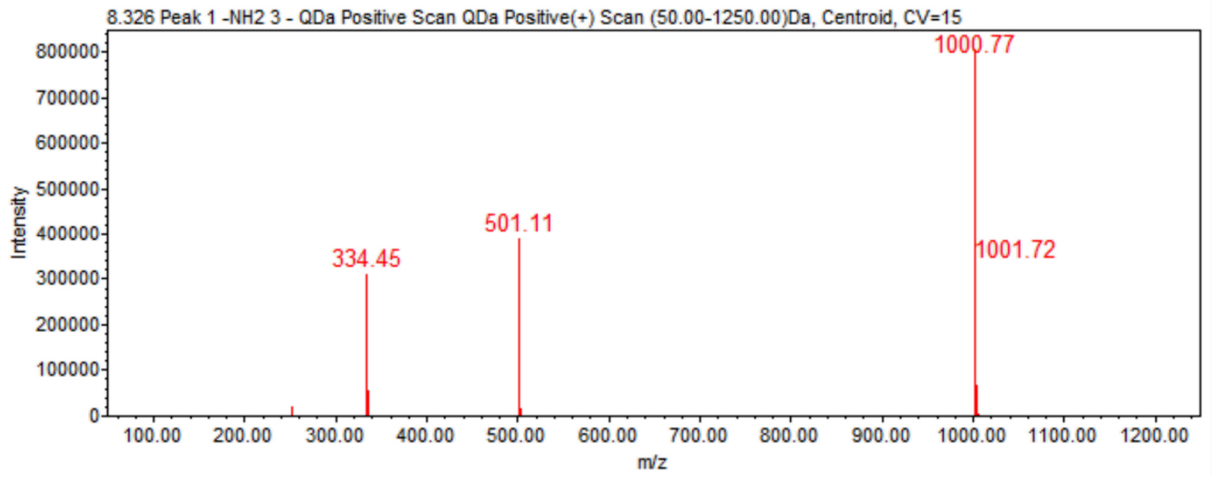

Figure S6. Mass spectrum of Pal-CKKRKC-NH_2.

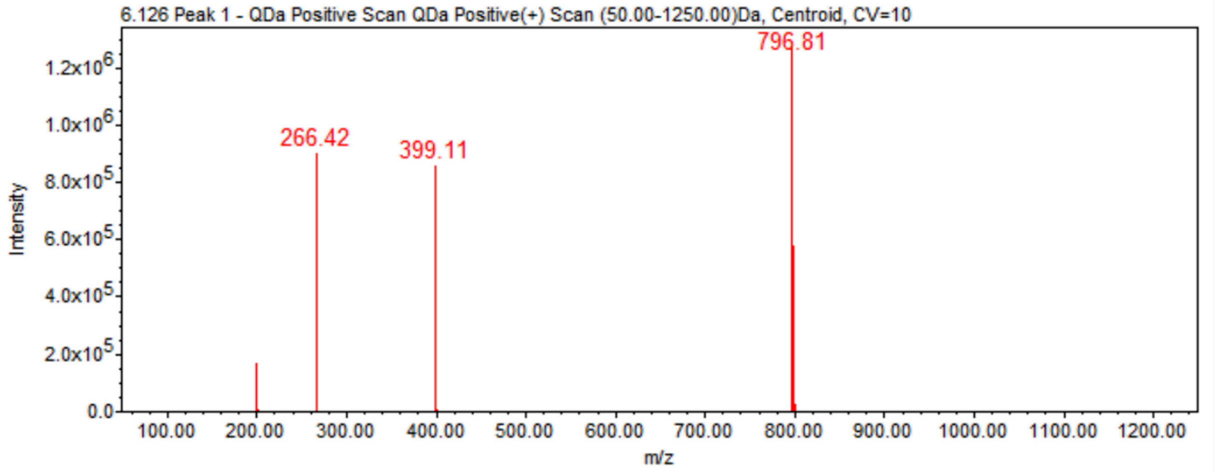

Figure S7. Mass spectrum of Pal-KKKR-NH_2.

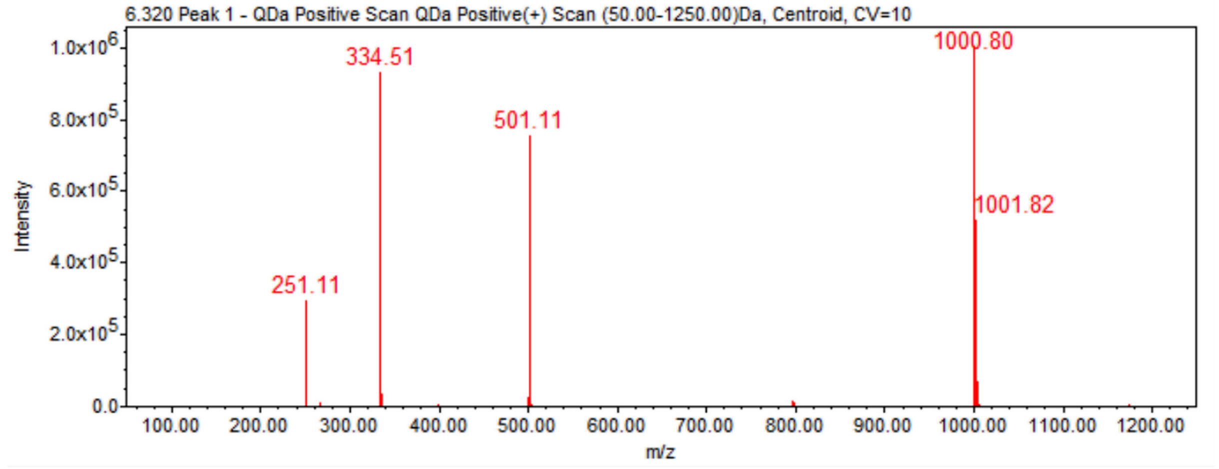

Figure S8. Mass spectrum of Pal-CKKKRC-NH_2 (cyclic).

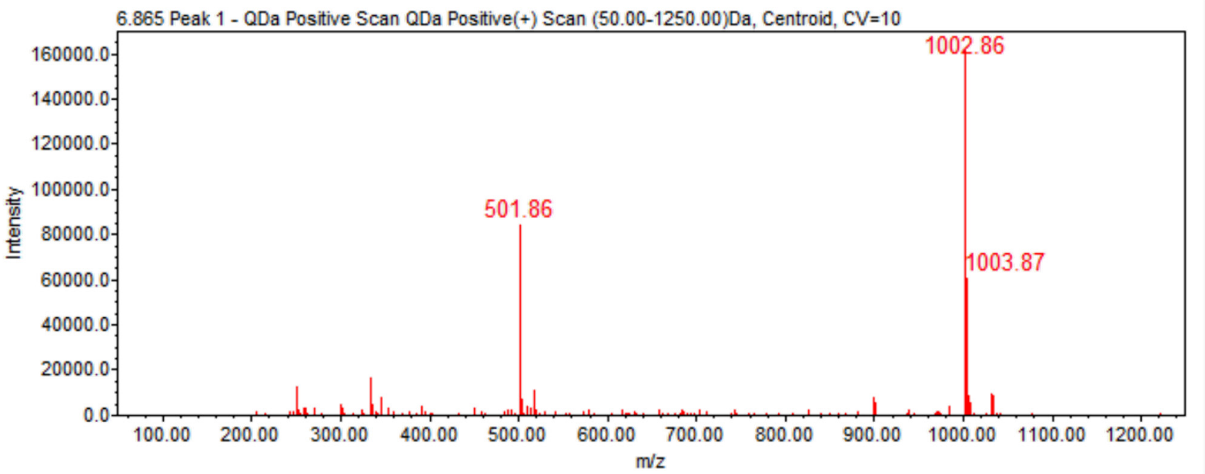

Figure S9. Mass spectrum of Pal-CKKKRC-NH_2 (linear).

© 2017 by the authors; licensee MDPI, Basel, Switzerland. This article is an open access article distributed under the terms and conditions of the Creative Commons by Attribution (CC-BY) license (http://creativecommons.org/licenses/by/4.0/).
